# QI project: Improvement in quality of Seclusion Medical Review

**DOI:** 10.1192/bjo.2021.584

**Published:** 2021-06-18

**Authors:** Shumaila Shahbaz, Richard Ward

**Affiliations:** Humber Teaching Foundation Trust

## Abstract

**Aims:**

To establish the improvements in the quality of seclusion medical review after introducing a template to complete the review.

**Background:**

The Mental Health Act – Code of Practice outlines the standards of patient care while in seclusion. It also emphasis that supportive engagement/observation schedules should be reviewed in person and continued at the point an episode of seclusion was initiated.

Furthermore, NICE also set up standards to monitor side effect profile while prescribing psychotropic for such patients and regular management review. It also gives importance to staff training to ensure these standards.

To improve the quality of the seclusion medical review, we completed an audit in July 2019 to ascertain whether medics are following Trust Policy.

We identified good results (above 90%) in the following areas:

Time of seclusion review

Record keeping

Management plan

Good documentation of risk, mental state examination and physical health.

We also noticed that the following areas can be improved:

Prescribed Medications. (60%)

Medication side effects. (40%)

Physical Observations (40%)

We used the following audit standards for our audit after our last audit and a template was designed and after discussion with medics incorporated into the existing documentation template.

Time of review

Reason and duration for seclusion

Psychiatric diagnosis

Mental State Examination/Behaviour

Physical health (including physical observations)/Environment

Medication (prescribed, rapid tranquilisation, side effects, or adverse effects)

Risk (to self-DSH or accidental) (risks to others)

Plan :(frequency of physical obs./medical review, management, restrictions, exit plan for terminating seclusion, patient's capacity to understand it)

**Method:**

We considered the following aspects:

Retrospective data collection from 01.03.2020 to 30.08.2020.

Sample selection: random selection of mixture of clinicians on different times and days of the week.

Data analysis was carried out by using Microsoft Excel.

**Result:**

We noticed a marked improvement in the quality of seclusion medical review (between 95% and 100%) after introducing a template for it. There were no major concerns identified during the re-audit.

**Conclusion:**

To continue to use the template for Seclusion Medical Review which has shown significant improvement in the quality of the reviews which will improve patient care.

It also helped us to deliver person centred care and safe practice.

To continue teaching and training of doctors.

This QIP project motivated nurses to do an audit on nursing seclusion review and made necessary changes.

